# Pembrolizumab-Induced Colitis With Concurrent Capecitabine in Triple-Negative Breast Carcinoma: A Case Report

**DOI:** 10.7759/cureus.101082

**Published:** 2026-01-08

**Authors:** Wesley Kafka, Wesam Frandah, John Diks

**Affiliations:** 1 Gastroenterology, Marshall University Joan C. Edwards School of Medicine, Huntington, USA; 2 Internal Medicine/Gastroenterology, Marshall University Joan C. Edwards School of Medicine, Huntington, USA; 3 Pathology, Marshall University Joan C. Edwards School of Medicine, Huntington, USA

**Keywords:** capecitabine, colitis, diarrhea, drug induced colitis, immune checkpoint inhibitors, immune mediated colitis, immune related adverse events, pd-1 inhibitor, pembrolizumab, triple negative breast cancer

## Abstract

Immune checkpoint inhibitors (ICIs) have revolutionized cancer treatment, but their use is associated with immune-related adverse events (irAEs). Colitis is a significant irAE, particularly with programmed cell death protein 1 (PD-1) inhibitors like pembrolizumab. This case report details a 48-year-old female with triple-negative breast cancer (TNBC) who developed colitis while on pembrolizumab and capecitabine. The patient presented with symptoms including diarrhea and stomatitis, which were initially managed with supportive care. Subsequent investigation with colonoscopy was normal appearing; however, biopsy revealed mild focal active colitis microscopically. This case highlights the importance of early recognition and management of pembrolizumab-induced colitis, especially in patients receiving concurrent chemotherapy, and emphasizes the need to differentiate it from other gastrointestinal issues or other drug-induced colitis, such as that caused by capecitabine. This case also adds evidence to help guide early histological identification of ICI-induced colitis, and a comprehensive literature review on pembrolizumab-induced colitis is presented, discussing its incidence, clinical presentation, diagnosis, and management strategies.

## Introduction

Immune checkpoint inhibitors (ICIs) have emerged as a cornerstone in modern oncology, offering significant therapeutic advancements across a wide spectrum of malignancies, including melanoma, non-small cell lung cancer (NSCLC), renal cell carcinoma, and triple-negative breast cancer (TNBC) [1,2]. These agents, such as pembrolizumab, function by blocking inhibitory immune checkpoints, like programmed cell death protein 1 (PD-1) or its ligand (PD-L1), thereby unleashing the host’s anti-tumor immune response [1,3]. While highly effective, this enhanced immune activity can inadvertently lead to immune-related adverse events (irAEs), which are essentially autoimmune or inflammatory reactions affecting various organ systems [1].

Among the diverse array of irAEs, colitis stands out as a particularly common and clinically significant complication of ICI therapy [1]. Pembrolizumab-induced colitis, though less frequent than with cytotoxic T-lymphocyte-associated protein 4 (CTLA-4) inhibitors, can range in severity from mild to life-threatening, necessitating prompt recognition and management to prevent severe outcomes such as bowel perforation or even death [1,2]. The incidence of pembrolizumab-induced colitis varies, with reported rates of moderate-to-severe cases affecting approximately 1% to 2% of patients [3]. The median time to onset of diarrhea associated with ICI-induced colitis is typically four to eight weeks after initiation of therapy, though symptoms can manifest earlier or much later [1,3].

Clinical presentation of ICI-induced colitis is often non-specific, encompassing symptoms such as diarrhea, abdominal pain, nausea, vomiting, and hematochezia [1]. Differentiating ICI-induced colitis from other causes of gastrointestinal distress, particularly infections like Clostridium difficile infection (CDI), is crucial for appropriate management [3]. Endoscopy with biopsy is considered the gold standard for diagnosis, revealing various histopathological patterns such as acute active colitis or microscopic colitis [1,2]. Management strategies are guided by the severity of colitis, often involving systemic corticosteroids, with second-line agents like infliximab reserved for steroid-refractory cases [1-3].

This case report discusses a unique presentation of pembrolizumab-induced colitis in a patient with triple-negative breast cancer receiving concurrent capecitabine. We highlight the diagnostic complexity of distinguishing between chemotherapy- and immunotherapy-induced diarrhea and emphasize the critical importance of early colonoscopy with random biopsies, as immune-mediated colitis may present with severe symptoms despite normal macroscopic findings. Furthermore, we provide a comprehensive review of the current literature on pembrolizumab-induced colitis, emphasizing its clinical features, diagnostic approaches, and therapeutic interventions to aid clinicians in optimizing patient care.

## Case presentation

A 48-year-old female with triple-negative breast cancer (TNBC) of the upper-outer quadrant of the left breast presented for an urgent visit with complaints of stomatitis, nausea, and diarrhea, along with sore hands and feet. Her initial diagnosis indicated clinically T2 N1 disease, with biopsy confirming triple-negative invasive ductal carcinoma. Her historical course included neoadjuvant chemotherapy with carboplatin, paclitaxel, doxorubicin, and cyclophosphamide in combination with pembrolizumab. A Mediport was placed prior to treatment initiation, and following neoadjuvant therapy, an ultrasound of the breast showed a complete response to treatment. The patient subsequently underwent lumpectomy and sentinel lymph node biopsy, which revealed residual disease. Post-surgery, the patient was recommended capecitabine at 1000 mg/m² twice daily, two weeks on and one week off, for eight cycles, referred to a radiation oncologist for further treatment, and pembrolizumab was continued as scheduled.

In the current presentation, laboratory reports were reviewed and remained stable (Table 1). Workup for infectious etiologies of diarrhea was negative. A Clostridioides difficile polymerase chain reaction (PCR) screen returned negative. Additionally, a multiplex gastrointestinal PCR panel was negative for common bacterial and viral pathogens, including Salmonella, Shigella, Campylobacter, Yersinia, Shiga toxin-producing E. coli, Norovirus, and Rotavirus. She was encouraged to continue magic mouthwash, alternate ondansetron and prochlorperazine, and continue loperamide as needed.

**Table 1:** Comprehensive laboratory results eGFR CKD-EPI: estimated Glomerular Filtration Rate calculated using the Chronic Kidney Disease Epidemiology Collaboration, ALT: Alanine aminotransferase, AST:  Aspartate aminotransferase, WBC: White blood cells, RBC: Red blood cells, MPV: Mean platelet volume, MCV: Mean corpuscular volume, MCH: Mean corpuscular hemoglobin, MCHC: Mean corpuscular hemoglobin concentration, RDW-CV: Red cell distribution width-coefficient of variation, RDW-SD: Red cell distribution width-standard deviation, NRBC: Nucleated red blood cell count, UA: Urinalysis.

Test	Result	Unit	Reference Range
Anion Gap	10	—	8–16
BUN	19	mg/dL	7–20
Creatinine	0.94	mg/dL	0.6–1.3
eGFR CKD-EPI	75	mL/min/1.73m²	≥60
Glucose (random)	144	mg/dL	70–140
Sodium	138	mEq/L	135–145
Potassium	2.8	mEq/L	3.5–5.1
Chloride	105	mEq/L	98–106
CO₂ (Bicarb)	23	mEq/L	22–29
Calcium	8.5	mg/dL	8.6–10.2
Magnesium	1.9	mg/dL	1.7–2.2
LIVER PANEL			
Test	Result	Unit	Reference Range
Albumin	3.9	g/dL	3.5–5.0
Total Protein	6.4	g/dL	6.0–8.3
Alkaline Phosphatase	106	U/L	44–147
ALT	39	U/L	7–56
AST	24	U/L	10–40
Total Bilirubin	1	mg/dL	0.2–1.2
CARDIAC/SPECIAL CHEMISTRY			
Test	Result	Unit	Reference Range
D-Dimer	1.1	mg/L	<0.5
Troponin-I (hs)	11	ng/L	<14
GENERAL HEMATOLOGY			
Test	Result	Unit	Reference Range
WBC	6.73	k/µL	4.0–11.0
RBC	4	M/µL	3.8–5.2
Hemoglobin	11.9	g/dL	12.0–16.0
Hematocrit	35.2	%	36–46
Platelets	250	k/µL	150–400
MPV	8.7	fL	9.0–12.5
RBC INDICES			
Test	Result	Unit	Reference Range
MCV	88	fL	80–100
MCH	29.8	pg	27–33
MCHC	33.8	g/dL	32–36
RDW-CV	15.9	%	11.5–14.5
RDW-SD	43.5	fL	39–46
MANUAL DIFFERENTIAL			
Test	Result	Unit	Reference Range
Neutrophils %	32.3	%	40–70
Neutrophils Absolute	3.18	k/µL	1.5–7.5
Bands	15	%	0–5
Lymphocytes %	10.1	%	20–40
Lymphocytes Absolute	0.68	k/µL	1.0–4.0
Monocytes %	27.3	%	2–10
Monocytes Absolute	1.84	k/µL	0.2–0.8
Eosinophils %	13.1	%	0–6
Eosinophils Absolute	0.88	k/µL	0.0–0.5
Basophils %	1	%	0–1
Basophils Absolute	0.07	k/µL	0.0–0.1
Immature Granulocytes %	0.4	%	0–0.5
Immature Granulocytes Abs	0.02	k/µL	0.0–0.03
NRBC	1	%	0
Myelocytes	1	%	0
MORPHOLOGY			
Test	Result	Unit	Reference Range
Anisocytosis	1+	—	None
Polychromasia	1+	—	None
Platelet Estimate	Normal	—	Normal
URINALYSIS DIPSTICK			
Test	Result	Unit	Reference Range
Urine Color	Yellow	—	Yellow
Urine Clarity	Turbid	—	Clear
UA Specific Gravity	1.027	—	1.005–1.030
UA pH	5.5	—	5.0–8.0
UA Protein	10	mg/dL	Negative–Trace
UA Glucose	Negative	—	Negative
UA Ketones	20	mg/dL	Negative
UA Blood	Negative	—	Negative
UA Bilirubin	Negative	—	Negative
UA Urobilinogen	Negative	mg/dL	0.2–1.0
UA Nitrite	Negative	—	Negative
UA Leukocyte Esterase	Negative	—	Negative
URINALYSIS MICROSCOPIC			
Test	Result	Unit	Reference Range
UA WBC	0–4	/HPF	0–5
UA RBC	0–2	/HPF	0–3
UA Squamous Cells	6–10	/HPF	0–5
UA Mucus	Present	—	None

**Figure 1:**
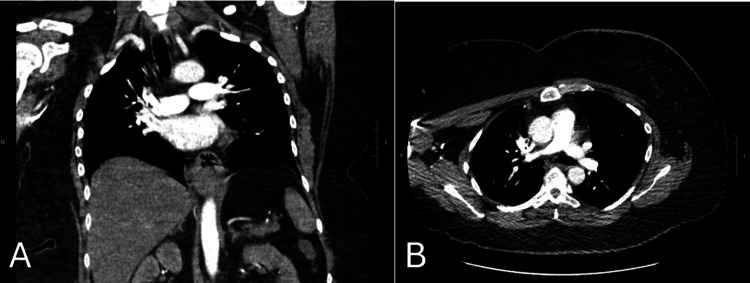
CT pulmonary angiography (A) Coronal and (B) Axial views with contrast opacification of the pulmonary arteries show no intraluminal filling defects.

The patient presented to the emergency department with left hip pain. Imaging showed no acute changes, but an elevated D-dimer (Table 1) prompted a CT angiogram of the chest, which showed no evidence of pulmonary embolism (Figure 1). She was discharged home on prednisone, and the following day, she received her next scheduled pembrolizumab treatment. She reported dysphagia and was treated with fluconazole for suspected fungal esophagitis, with a referral to the gastrointestinal (GI) clinic for further evaluation. As such, the capecitabine dose was planned to be reduced from 2000 mg to 1500 mg twice daily, with a new cycle starting. 

At one-month follow-up, laboratory evaluation demonstrated hypokalemia. Despite potassium replacement, significant adverse effects persisted, and capecitabine was therefore held. Dihydropyrimidine dehydrogenase enzyme testing was ordered, which later returned normal. Two weeks later, she reported that hand-foot syndrome and diarrhea had almost completely resolved, and she continued radiation therapy. Pembrolizumab was continued, and the patient was restarted on capecitabine at a reduced dose of 1500 mg twice daily, with two weeks on and one week off. After one month, an esophagogastroduodenoscopy and colonoscopy were performed, and biopsies revealed mild focal active colitis in the colon, with no evidence of dysplasia. On follow-up, the patient was tolerating the current treatment well, with the capecitabine dose reduction and supportive measures proving beneficial. 

**Figure 2:**
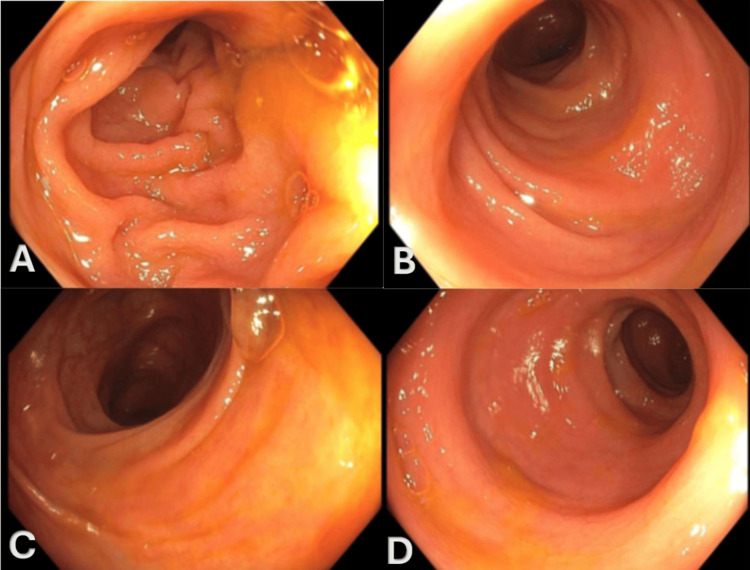
Colonoscopy images Colonoscopy demonstrating no evidence of bleeding, ulceration, or inflammation in the transverse (A), descending (B), ascending (C), and sigmoid colon (D).

Throughout this period, the patient experienced persistent diarrhea, raising suspicion for drug-induced colitis. Based on the persistence of symptoms and the requirement for systemic corticosteroid intervention, the adverse event was classified as Common Terminology Criteria for Adverse Events (CTCAE) Grade 2 colitis. Upper gastrointestinal endoscopy and colonoscopy were performed, revealing no gross evidence of ulceration, inflammation, or malignancy (Figure 2). Given the high clinical suspicion for immunotherapy-induced colitis and the potential for microscopic inflammation often associated with checkpoint inhibitors, random biopsies were obtained despite the normal macroscopic appearance.

**Figure 3:**
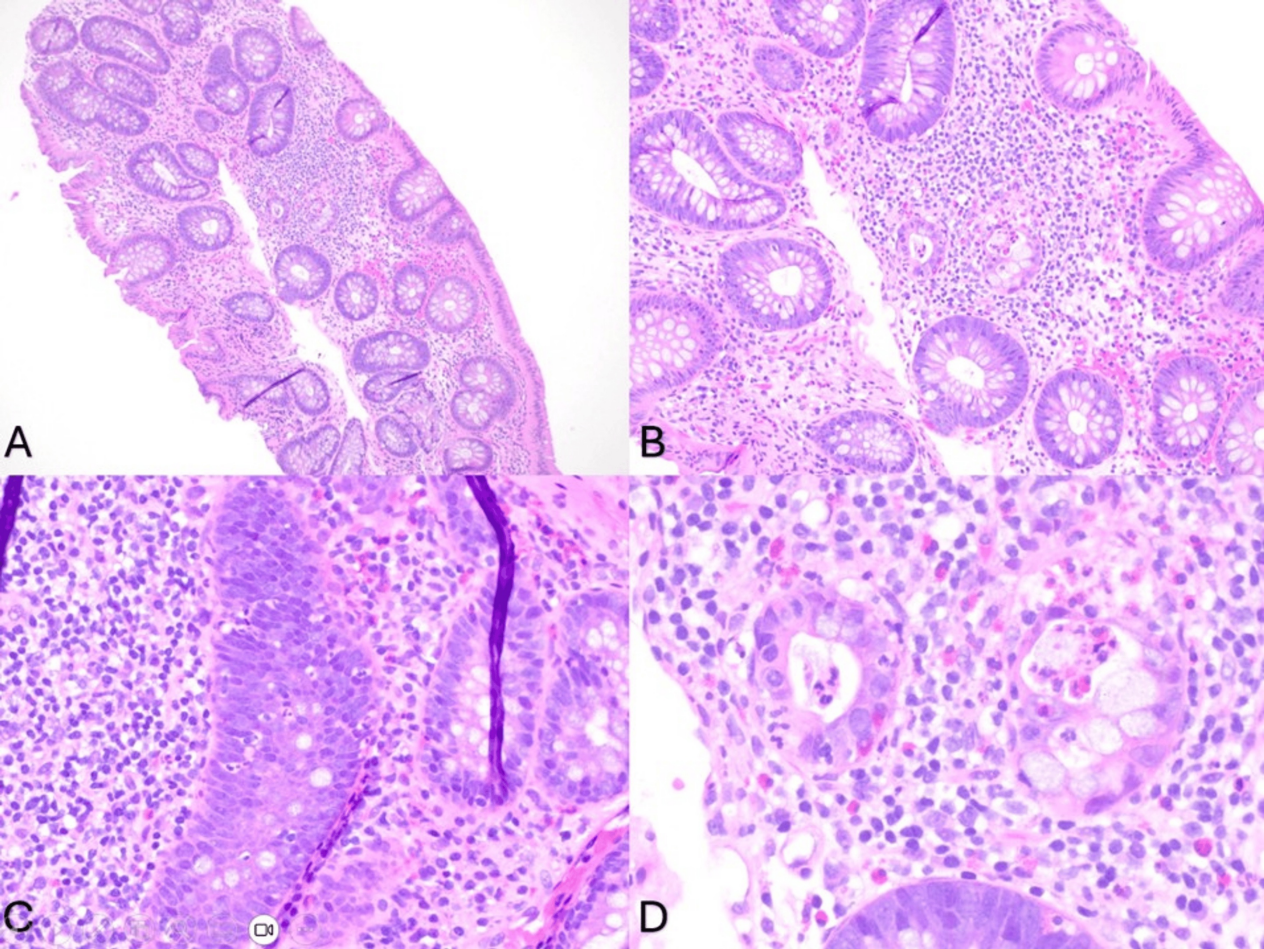
Histopathology images (A) Mild focal active colitis. Intact colonic mucosa with diffuse lymphocytic and plasma cell infiltration (H&E, 100X). (B) Mild focal active colitis. Intact colonic mucosa with diffuse lymphocytic and plasma cell infiltration (H&E, 200X). (C) Mild focal acute colitis. Intact colonic mucosa with diffuse lymphocytic and plasma cell infiltration. Rare neutrophils and eosinophils were noted within crypts and scattered throughout (H&E, 400X). (D) Mild focal acute colitis. Intact colonic mucosa with diffuse lymphocytic and plasma cell infiltration. Increased visualization of neutrophils and eosinophils (H&E, 600X).

Upper GI biopsies showed preserved villous architecture and mild chronic inactive inflammation without evidence of Celiac disease or Helicobacter-like organisms, and esophageal biopsies were unremarkable. In contrast, the colon biopsies revealed mild focal active colitis, consistent with an immune-related adverse event secondary to pembrolizumab (Figure 3). The patient’s symptoms were managed with supportive care and dose adjustments as described. The histopathological confirmation of colitis, particularly in the setting of persistent symptoms despite holding capecitabine, supported the diagnosis of pembrolizumab-induced colitis.

This case underscores the importance of vigilant monitoring and the necessity of histological evaluation in patients receiving ICI therapy, particularly when gastrointestinal symptoms arise. Table 2 shows the clinical timeline of oncology treatment and colitis presentation.

**Table 2:** Clinical timeline of oncology treatment and colitis presentation TNBC: Triple-negative breast cancer, BID: Twice in a day, PE: Pulmonary embolism, ER: Emergency room, GI: Gastrointestinal, ASCO/NCCN: American Society of Clinical Oncology/National Comprehensive Cancer Network.

Phase	Event / Intervention	Clinical Details
Initial Diagnosis	Presentation	48-year-old female diagnosed with TNBC (cT2 N1).
Neoadjuvant	Chemo-Immunotherapy	Carboplatin, paclitaxel, doxorubicin, cyclophosphamide+pembrolizumab.
Surgery	Lumpectomy & SLNB	Ultrasound showed a complete response; Pathology showed residual disease.
Adjuvant Start	Concurrent Therapy	Capecitabine (1000 mg/m² BID, two weeks on/one week off) + Pembrolizumab (continued).
Symptom Onset	Toxicity Presentation	Developed stomatitis, nausea, diarrhea, and sore hands/feet (Palmar-plantar erythrodysesthesia).
Management	Initial Supportive Care	Magic mouthwash, antiemetics, loperamide. Lab results stable.
Acute Event	ER Visit	Left hip pain. D-dimer elevated. CT Angiography chest negative for PE. Discharged on prednisone.
Progression	Continued Therapy	Received scheduled pembrolizumab dose following ER discharge.
Worsening	New Symptoms	Developed dysphagia (suspected fungal esophagitis). Treated with fluconazole.
Intervention 1	Dose Adjustment	Capecitabine dose reduced (2000 mg to 1500 mg daily). Referred to the GI clinic.
Diagnostic	Workup	Stool infectious panel negative. Colonoscopy performed (grossly normal).
Diagnosis	Histopathology	Random biopsies revealed focal active colitis consistent with immune-mediated toxicity.
Intervention 2	Final Management	Pembrolizumab held. Systemic corticosteroids continued/tapered per ASCO/NCCN guidelines.

## Discussion

Immune checkpoint inhibitors (ICIs), particularly PD-1 inhibitors like pembrolizumab, have revolutionized cancer therapy by enhancing the host’s anti-tumor immune response. Despite these advancements, immune-mediated responses via T cell overactivation lead to various adverse events, including colitis. Pembrolizumab has the lowest incidence rates of colitis and diarrhea compared to other ICIs, but remains a notable and potentially severe complication [1]. Our patient, a 48-year-old female with TNBC, developed symptoms consistent with colitis while on pembrolizumab, ultimately confirmed by biopsy as mild focal active colitis.

Incidence and clinical presentation 

Pembrolizumab-induced colitis typically develops within four to eight weeks of ICI initiation, though onset can vary widely [1,3]. Contrast this to capecitabine-associated colitis, which usually starts with diarrhea two to three weeks after initiation. The incidence of moderate-to-severe colitis with pembrolizumab is reported to be between 1% and 2% [3]. Clinical manifestations are often non-specific and can include diarrhea, abdominal pain, nausea, vomiting, and hematochezia [1]. Our patient experienced diarrhea, nausea, and stomatitis, which are common symptoms. It is crucial to differentiate ICI-induced colitis from other causes of gastrointestinal symptoms, such as infections (e.g., Clostridioides difficile), inflammatory bowel disease, or tumor metastasis [1,3]. The case presented by Zhou et al. highlights the challenge of concurrent Clostridioides difficile infection (CDI) and pembrolizumab-induced colitis, where symptoms worsened despite CDI treatment, leading to suspicion of irAE-colitis [3]. Similarly, Sisman et al. reported a fatal case of pembrolizumab-induced colitis with concurrent Giardia infection, emphasizing the need for thorough investigation [2]. In our patient’s case, infectious etiologies were considered and ruled out through clinical assessment and microbiology results. 

Diagnosis 

The gold standard for diagnosing ICI-induced colitis is endoscopy with biopsy. This should be considered in patients receiving ICI therapy with grade 2 or higher diarrhea, defined as an increase of 4-6 stools/day over baseline, a moderate increase in ostomy output over baseline, or limiting instrumental activities of daily living [1,2,4]. Our patient did not present with grade 2 diarrhea, but a colonoscopy was still performed due to the combination of capecitabine and pembrolizumab. This raises the question of how to address patients receiving multiple agents known to induce colitis. In our case, the patient’s symptoms were almost completely resolved but remained despite discontinuation of capecitabine and a normal appearing colonoscopy. The histopathological examination of the biopsy ensured early recognition and diagnosis of colitis while also differentiating the type prior to restarting the reduced dose of capecitabine. 

Endoscopic findings in immune checkpoint inhibitor (ICI)-induced colitis are highly variable, ranging from normal-appearing mucosa to severe edema and ulceration [1]. This variability underscores the critical importance of obtaining random biopsies even in the absence of macroscopic abnormalities. Histopathological evaluation plays a decisive role in the differential diagnosis, particularly when distinguishing between immune-mediated toxicity and chemotherapy-induced injury. ICI-induced colitis typically presents with patterns of acute or chronic active colitis, characterized by neutrophilic infiltration, cryptitis, crypt abscesses, and increased epithelial apoptosis [2,5-7].

This contrasts distinctively with fluoropyrimidine-associated mucositis (e.g., secondary to capecitabine), which results from direct cytotoxicity. Chemotherapy-induced changes typically manifest as crypt dilation, epithelial atypia, mucin depletion, and architectural disarray (such as dropout or atrophy), often with relatively sparse inflammation or a neutrophilic predominance lacking the lymphocytic component seen in immune reactions. In our case, the patient’s biopsy revealed mild focal active colitis with predominant crypt lymphocytic infiltration and background lamina propria eosinophils (Figure 2). Crucially, the preservation of crypt architecture helped exclude chronic inflammatory bowel disease (IBD), while the absence of viral cytopathic effects (e.g., CMV inclusions) ruled out infectious etiologies. Therefore, the presence of active inflammatory changes without the architectural atypia characteristic of cytotoxic damage strongly supported the diagnosis of pembrolizumab-induced colitis [2,5-7]. 

Another histopathological issue is the understanding of early markers of ICI-induced colitis and how these findings further differentiate this from other forms of drug-induced colitis. These ICI drugs increase the activity of T-cell activation, leading to widespread lymphocytic activity; however, infiltration of neutrophils and, more uniquely, eosinophils may mark early presentations of ICI colitis [8]. Eosinophils are noted to be found in the intestinal lamina propria with ranges of 20-30 and even up to 50 per high-power field. However, colonic findings are more scarce, with the proximal colon showing occasional eosinophils and the distal colon being absent of these cells in normal tissue [9]. Another important distinguishing factor is age, where in adults, the normal range demonstrates 1-3 eosinophils per high-powered field compared to 50-100 in children [10]. The findings in our patient show increased infiltration, as shown in Figures 4, 5. There is little evidence in this area, but this is consistent with a case report by Markovic et al., which underscores the importance of early identification and support in diagnosing [8]. 

The potential utility for assessment of eosinophils relates to its role as a marker for inflammation, whether circulating or in direct tissue such as the colon [11,12]. These cells may be an early predictor for irAEs induced by checkpoint inhibitors, and although inflammatory toxicities may be seen in various tissues or organs, they occur more commonly in barrier organs, including the gastrointestinal tract, with specific emphasis on the colon [13,14]. Additionally, despite case reports of capecitabine-induced colitis, there is a markedly increased association with involvement in the ileum, where there is a greater significance of eosinophilic infiltration as well as gross changes of the intestine [15].

Management 

The management of this patient, including the interruption of pembrolizumab and initiation of corticosteroid therapy, was consistent with current clinical practice guidelines established by the American Society of Clinical Oncology (ASCO) and the National Comprehensive Cancer Network (NCCN) for the management of immune-related adverse events. Both guidelines recommend the suspension of immunotherapy and the administration of systemic corticosteroids (e.g., prednisone 1-2 mg/kg/day) for patients presenting with Grade 2 or higher immune-mediated colitis [16,17].

Management of ICI-induced colitis is guided by the severity of symptoms, typically graded using the Common Terminology Criteria for Adverse Events (CTCAE) [1,2]. For mild symptoms (Grade 1), supportive care, such as hydration and dietary modifications, may suffice [3]. Moderate to severe cases (Grade 2 or higher) usually require systemic corticosteroids [1,3]. Our patient’s symptoms, while bothersome, were managed with supportive measures and dose adjustments of capecitabine, which also contributed to gastrointestinal side effects. The incomplete resolution despite improvement in her symptoms after capecitabine dose reduction and continued pembrolizumab, along with the histopathological biopsy findings, supported the diagnosis of pembrolizumab-induced colitis. For steroid-refractory cases, second-line agents such as infliximab, a tumor necrosis factor (TNF)-α inhibitor, are often employed [1-3]. Vedolizumab may be considered for cases refractory to infliximab [2]. Early recognition and appropriate intervention are critical to prevent severe complications like bowel perforation and to allow for continued ICI therapy if possible [1,2].

## Conclusions

This case demonstrates the successful resolution of pembrolizumab-induced colitis in a patient with triple-negative breast cancer following the discontinuation of immunotherapy and the initiation of corticosteroids. The clinical course highlights the diagnostic challenge of distinguishing immune-mediated colitis from chemotherapy-induced diarrhea, particularly when concurrent capecitabine is used. Crucially, this report reinforces the necessity of performing a colonoscopy with random biopsies even when the mucosa appears macroscopically normal. Reliance on visual endoscopic findings alone may lead to misdiagnosis; therefore, histopathological differentiation remains the gold standard to guide appropriate management and prevent the premature withdrawal of essential concurrent chemotherapies. While routine colonoscopy is not indicated for all cases of mild diarrhea, early endoscopic evaluation is recommended for patients presenting with diagnostic uncertainty, particularly those receiving concurrent diarrheogenic agents or those failing to respond to standard anti-motility agents. In these selected high-risk scenarios, early visualization and biopsy are critical to rule out immune-mediated toxicity that may otherwise be masked by attribution to chemotherapy.
